# Changes in biodiversity drive trypanosome infections of wildlife in Panama

**DOI:** 10.1016/j.onehlt.2025.101113

**Published:** 2025-06-18

**Authors:** Magdalena Meyer, Georg Eibner, Alexander Christoph Heni, Kerstin Wilhelm, Simone Sommer

**Affiliations:** Institute of Evolutionary Ecology and Conservation Genomics, Ulm University, Ulm, Germany

**Keywords:** Zoonotic diseases, Anthropogenic disturbance, *Trypanosoma cruzi* infections, Diversity-disease relationship, Biodiversity conservation, Host community composition, Genetic diversity, One health

## Abstract

Understanding how anthropogenic disturbances impact biodiversity and zoonotic disease transmission is critical for safeguarding human and animal health. In Panama, we studied the effects of these disturbances on wildlife populations and *Trypanosoma cruzi* infections, which cause Chagas disease in humans, at 23 different sites ranging from pristine forests to heavily altered monocultures. Our results indicate that human disturbance leads to increased trypanosome infection rates, primarily through two mechanisms: the proliferation of generalist marsupial host species, specifically *Didelphis marsupialis* and *Philander opossum*, which are key reservoirs for *Trypanosoma cruzi*, and a decline in the genetic diversity of the alternative rodent host *Proechimys semispinosus*. While species diversity did not affect infection probability in protected habitats, where natural processes support ecological resilience, higher diversity in disturbed, unprotected habitats was linked to a reduced risk of infection. These findings highlight the consequences of human impacts on wildlife diversity, including species assemblages and genetic diversity, and their potential role in disease ecology. We emphasize that conservation of pristine ecosystems and natural species communities is essential for mitigating zoonotic disease risks and preserving ecosystem health.

## Highlights

• Human disturbances increase trypanosome infections by altering wildlife communities.
• Resilient generalist host species are thriving in disturbed habitats and display a higher trypanosome prevalence.
• In host species, reduced genome-wide genetic diversity in disturbed landscapes is linked to higher infection rates.
• Habitat protection and species conservation help mitigate the risks of zoonotic disease transmission.

## Graphical abstract

This graphical abstract was created with BioRender.com.

**Figure:**
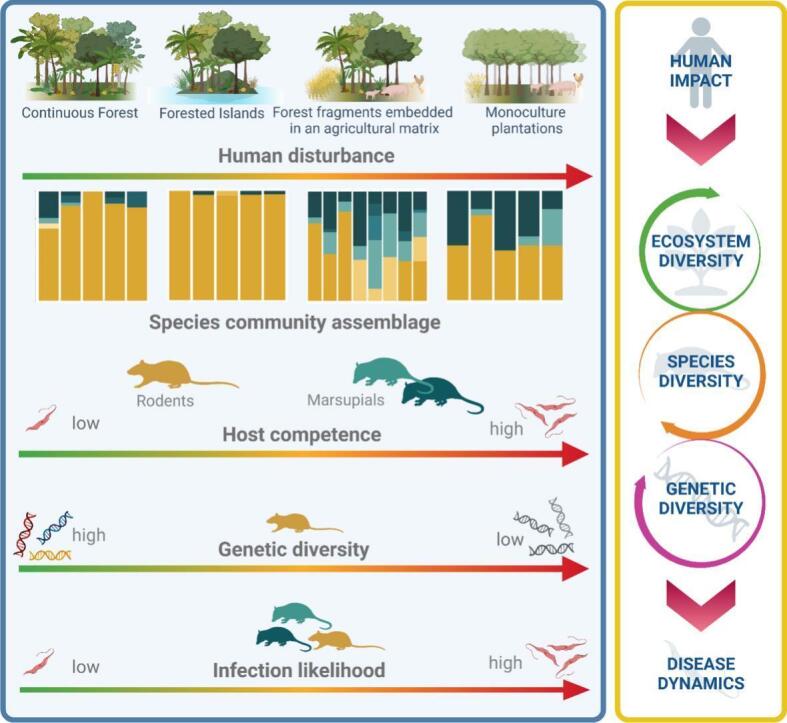

## 1 Introduction

Biodiversity, encompassing ecosystem diversity, species diversity, and genetic variability, serves as a cornerstone for ecosystem health and stability [[1], [2], [3], [4], [5], [6], [7]]. Yet, unprecedented global extinction rates—largely driven by human activities breaching planetary boundaries—have precipitated a severe biodiversity crisis [8,9]. As biodiversity declines due to habitat destruction, deforestation, fragmentation, and other human-induced disturbances, these ecological changes can trigger cascading effects that reverberate through ecosystems with significant consequences for health [5,[10], [11], [12], [13]]. The link between biodiversity and health is complex and multifaceted. In addition to providing essential services like clean air and water, soil fertility, and climate regulation—all of which are vital to human well-being [[14], [15], [16], [17]]—biodiverse ecosystems also play a critical role in shaping disease dynamics [[18], [19], [20]]. Changes in wildlife communities, such as shifts in species assemblages, can alter ecological interactions and potentially affect the transmission and spread of zoonotic pathogens [[21], [22], [23]]. Depending on the nature of these interactions and the characteristics of the host community, such changes may either amplify or reduce pathogen transmission [[22], [23], [24], [25], [26], [27], [28], [29], [30]]. These dynamics highlight the complex connections between biodiversity and disease ecology, where disruptions to natural systems can have cascading effects on both ecosystem and health outcomes. Recognizing these intricate connections, the One Health framework highlights the interdependence of environmental, animal, and human health [31].

Such an approach is particularly relevant for neglected tropical diseases (NTDs), which often emerge in the context of early-stage ecosystem degradation and disproportionately affect vulnerable populations in tropical and subtropical regions [32,33]. Environmental changes, such as climate warming, deforestation, and other forms of land-use transformation, are especially pronounced in biodiverse regions and can undermine natural disease regulation mechanisms that help buffer against pathogen spillover [[34], [35], [36]]. These effects are most acutely felt in socioeconomically marginalized communities, where limited access to healthcare further exacerbates disease burdens [32,37]. Among these NTDs, Chagas disease (American Trypanosomiasis) is of particular concern due to its significant impact on public health [[38], [39], [40]]. Chagas disease, impacting approximately 6–7 million people globally, primarily in Latin America, is often referred to as ‘silent and silenced’ due to its slow progression and its prevalence among marginalized populations who lack political representation and access to healthcare [32,40,41]. However, by now approximately 75 million people are considered at risk of infection [42]. Once limited to rural areas of the Americas, Chagas disease has now spread to 44 countries, including Canada, the United States, and parts of Europe, largely due to migration of infected individuals into non-endemic regions [43]. Although vectorial transmission remains restricted to the Americas because triatomine vectors are absent elsewhere, climate change and environmental alterations may create favorable conditions for these vectors in temperate areas, such as southern Europe (e.g., Portugal, Spain, and Italy) and parts of Australia [44]. Often asymptomatic in its early stages, Chagas disease can lead to severe morbidity, with up to 30 % of chronically infected individuals developing cardiac or gastrointestinal complications that may result in disability or death if left untreated. While antiparasitic treatments are available and most effective during the acute phase, their efficacy diminishes over time, and no vaccine currently exists [39,45]. Chagas disease is a vector-borne illness caused by the protozoan parasite *Trypanosoma cruzi*, mainly transmitted through the feces or urine of triatomine insects, commonly known as kissing bugs [39,46]. These insects are highly adaptable generalists capable of infecting a wide range of host species [47]. The transmission of Chagas disease involves a complex host-pathogen system, where various species of mammals serve as both hosts and reservoirs for *Trypanosoma cruzi* [48]. In fact, more than 180 *Trypanosoma cruzi* host species within seven mammalian orders have been identified so far [49]. Despite advances in our understanding and efforts to control Chagas disease, achieving sustainable disease management remains a significant challenge, partly due to the enormous host diversity [50]. Although recent studies have begun to examine the influence of climate change, vegetation cover, and land-use transformation on *Trypanosoma cruzi* transmission, our understanding of how these factors shape disease dynamics in wildlife, particularly in terms of changes in the distribution, composition, and diversity of host communities, is still limited [[51], [52], [53]].

In this study, we investigated the effects of anthropogenic disturbances on the dynamics of *Trypanosoma cruzi* infections within wild small mammal communities. We conducted a comprehensive investigation across a gradient of human impact and land-use, from protected, pristine lowland rainforests to highly fragmented commercial monoculture plantations within an agricultural matrix in Panama. Our study examines how changes in species community assemblages and host genetic diversity relate to trypanosome infection patterns. By comparing landscapes with varying levels of protection, we demonstrate the influence of land-use change and conservation efforts on pathogen transmission. These findings offer novel insights that may inform both disease management and biodiversity conservation strategies.

## 2 Material and methods

### 2.1 Study area, small mammal trapping and sample collection

**Fig. 1:**
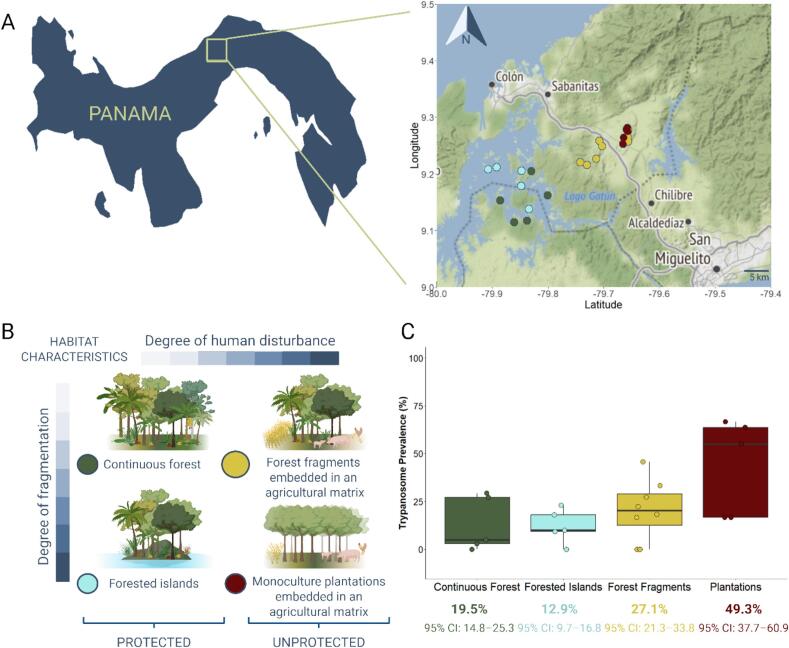
Study area, landscape types, and trypanosome prevalence in Panama. Study area in central Panama with location of the sampling sites (A), belonging to different landscape types (B). The trypanosome prevalence of all captured small mammals (n = 830) across the four different landscape types (C). Each data point in the boxplots represents prevalence at an individual capture site, while the values below each boxplot indicate overall prevalence per landscape, with 95 % confidence intervals. Map was created with OpenStreetMap and icons were created with BioRender.com.

The study was carried out in central Panama over the course of two fieldwork seasons (October 2013–May 2014 and October 2014–May 2015), which spanned the time of high animal activity, the transition from the rainy season to the dry season. The region is characterized by a tropical climate, with uniform conditions across all study sites. In order to minimize temporal bias, the order of capture sites was alternated between seasons. A total of 830 small mammals were live-trapped across 23 study sites in the Panama Canal area located in four different landscapes that differ in their degree of human disturbance and fragmentation. Multiple study sites were investigated within each landscape (see Fig. 1, for details see Schmid et al., 2018 [54]. The landscape referred to as “Continuous Forest” surrounding Lake Gatun, represented a pristine continuous lowland rainforest, exhibiting the least amount of human influence and disturbance. The landscape “Forested Islands” consisted of forested islands within the Panama Canal zone. These islands were once former hilltops, which became isolated after the Panama Canal area was flooded approximately a century ago. The present-day terrestrial fauna on these islands likely descends from species that inhabited the region prior to this isolation. These islands represent a fragmented protected habitat without any additional anthropogenic disturbance. For over 70 years, the sites located in the lowland continuous forest and on the islands have been safeguarded and preserved under the protection of the Barro Colorado Nature Monument (BCNM) and no humans (except for security and research) as well as no domestic animals are permitted. Contrarily, the study sites in the further two landscapes, represented by forest fragments and plantations were located in an area experiencing significant land-use changes and population growth over the past 60 years due to the construction of the Transístmica highway in the 1950s [55]. "Forest Fragments", an agricultural landscape, consisted of small forest fragments embedded in an agricultural matrix. "Monoculture Plantations" refers to areas characterized by intense land-use practices, predominantly consisting of monocultural tree plantations, primarily teak (*Tectona grandis*) and occasionally spiny cedar (*Pachira quinata*). In each landscape, a minimum of five study sites were investigated, each serving as an independent replicate. Each study site was sampled once per field season over five consecutive nights using 100 evenly spaced trapping stations arranged at 20-m intervals along parallel lines. Each station included three traps—two ground traps (a Tomahawk trap measuring 15.2 cm × 15.2 cm × 48.3 cm and a Sherman trap measuring 10.2 cm × 11.4 cm × 38.1 cm) and one Sherman trap set 0.5–2.5 m above ground on a liana or tree branch where feasible—resulting in a total of 300 traps per study site. For sites where it was not possible to accommodate all 100 trapping stations due to size constraints (landscape A: site AS4, landscape I: sites MG and GU), adjustments were made in subsequent analyses to account for the lower number of trap events. Traps were opened at dusk and baited with a mixture of peanut butter, oatmeal, bird seed, banana, and small pieces of dry dog food to attract species with a range of dietary preferences, and were checked and closed at dawn the following morning. For trypanosome screening, minimally invasive blood samples (10–75 μl) were collected from a small ear cut or caudal vein venipuncture using EDTA-coated capillaries (75 μl, Kabe, Germany) and stored at −80 °C until analysis. Further information regarding animal handling can be found in Schmid et al., 2018 [54]. The research conducted in this study obtained full ethical approval from the Smithsonian IACUC (Institutional Animal Care and Use Committee) protocols 2013-0401-2016-A1-A7 and 2016-0627-2019-A1-A2. The samples were legally exported to Germany with the permission of the Panamanian Government, specifically under permit numbers SE/A-21-14–SE/A-12-18, SE/A-69-14, SEX/A-22-15, SEX/A-24-17, SEX/A-120-16, and SEX/A-52-17.

### 2.2 *Trypanosoma* screening

DNA was extracted from blood samples (*n* = 830) using the MagNAPure 96 DNA and Viral DNA Small Volume Kit (Roche, Switzerland). In order to detect trypanosomatid infections, we used a nested PCR approach targeting a conserved region of the 18S rRNA gene, following Noyes et al. [56]. The first round of PCR used primers TRY927F (5′-GAAACAAGAAACACGGGAG-3′) and TRY927R (5′-TACTGGGCAGCTTGGA-3′) to amplify a 927 bp fragment. The PCR conditions included a reaction volume of 10 μl, consisting of 1 μl DNA extract, 5 μl DreamTaq Mastermix, 3.4 μl H_2_O and 0.3 μl of each primer solution (≙ 3 pmol). Thermocycling conditions began with denaturation at 95 °C for 2 min, followed by 35 cycles of 95 °C for 30 s, primer annealing at 59.5 °C for 60 s, and elongation at 72 °C for 45 s, concluding with a final elongation at 72 °C for 10 min. PCR products were run on a 1.5 % agarose gel in TAE buffer at 100 V for 60 min. Gels were visualized under UV light, and samples showing bands of the expected size (∼900 bp) were used as templates in a second PCR with internal primers SSU561F (5′-TGGGATAACAAAGGAGCA-3′) and SSU561R (5′-CTGAGACTGTAACCTCAAAGC-3′). The reaction volume was again 10 μl, using 2 μl of the first PCR product as a template with 5 μl DreamTaq Mastermix, 3.4 μl H2O, and 0.3 μl of each 10 pmol/μl primer solution (≙ 3 pmol). Thermocycling conditions were identical to the first PCR round, with primer annealing adjusted to 55 °C. The resulting amplicons (∼600 bp) were selected for another PCR approach with primers 121 (5′-AAATAATGTACGGGTGAGATGCATGA-3′) and 122 (5′-GGTTCGATTGGGGTTGGTGTAATATA-3′) that targeted a highly conserved region in kinetoplastoid DNA minicircles producing ∼330 bp bands for *Trypanosoma cruzi* infections [57,58]. Each 10 μl PCR reaction contained 1 μl DNA extract, 5 μl DreamTaq Mastermix, 3.4 μl water, and 0.3 μl of each 10 pmol/μl primer solution (≙ 3 pmol). Thermocycling conditions began with an initial denaturation at 95 °C for 3 min, followed by 35 cycles of denaturation at 95 °C for 45 s, primer annealing at 63 °C for 45 s, and elongation at 72 °C for 45 s, with a final elongation at 72 °C for 10 min. Products were visualized on a 1.5 % agarose gel in TBE buffer, run at 100 V for 45 min. All samples that displayed a ∼ 330 bp band visible under UV light were considered positive for *Trypanosoma cruzi*. Each PCR included one positive and one negative control to ensure reliability of the results.

### 2.3 Genetic diversity analysis

As part of one of our previous studies, SNP genotyping was employed to assess the genomic diversity of the predominant small mammal in our study, *Proechimys semispinosus*, also known as the Tome's spiny rat [59]. To this end, DNA was extracted from ear tissue samples from a representative subset of overall 262 individuals (N_Continuous Forests_ = 108, N_Forested Islands_ = 70, N_Forest Fragments_ = 54, N_Plantations_ = 30) using the NucleoSpin® 96 Tissue Kit (Macherey-Nagel). Genotyping-by-sequencing (GBS) library preparation was performed using the restriction enzyme *Mse*I, followed by paired-end 150 bp sequencing on an Illumina NextSeq 500 V2 platform, yielding approximately 3 million reads per individual (LGC Genomics GmbH). SNP calling was performed using the de novo pipeline of STACKS v2.2 [[60], [61], [62], [63]]. We applied filtering criteria, including a minimum minor allele frequency of 0.1, a maximum observed heterozygosity of 0.95, and SNP presence in at least 85 % of individuals and in at least 10 study sites. To reduce linkage effects, we retained only the first SNP per locus in the analysis. Loci potentially under positive selection were identified and excluded using BayeScan [64], resulting in a final dataset of 6917 presumably selectively neutral SNP loci. To characterize genomic diversity at the individual level, we used VCFtools v0.1.15 [65] to calculate the individual inbreeding coefficient (F) as an indicator of individual heterozygosity. Values were inverted (1 - inbreeding coefficient F) so that higher values corresponded to greater individual genomic diversity. For comprehensive information regarding the sequencing approach, bioinformatic analysis and results, please refer to Schwensow et al., 2022 [59].

### 2.4 Statistical analysis

All analyses were performed using the software R (v4.1.1; R Core Team 2022 [66]). To estimate species diversity in the small mammal communities, the Shannon index was calculated for each capture site. Differences in species community composition between the landscapes were assessed using a Permutational Multivariate Analysis of Variance (PERMANOVA) via the adonis2() function in the ‘vegan’ package, based on a Jaccard dissimilarity matrix computed from presence–absence data [67]. Statistical significance was evaluated using 999 permutations, and post hoc pairwise comparisons were performed with pairwise.perm.manova() from the ‘RVAideMemoire’ package, applying Holm correction to adjust *p*-values for multiple comparisons [68]. Population density of the generalist species, the marsupials *Didelphis marsupialis* and *Philander opossum,* and the rodent *Proechimys semispinosus* were calculated for each capture site by dividing the number of captured individuals by the area that was covered by the traps. Trypanosome prevalence was calculated for each species and infraclass as well as for each landscape using the ddply() function in the ‘plyr’ package [69]. For all prevalence estimates, 95 % confidence intervals (CI) were calculated using the Wilson method with the binom.confint() function from the ‘binom’ package [70]. Fisher's Exact Test was used for analyzing differences in trypanosome prevalence among the different host species. To assess differences in trypanosome prevalence across landscapes, we used a binomial Generalized Linear Model (GLM) with a logit link function, modeling the number of infected and uninfected individuals per capture site. Post hoc comparisons between landscapes were performed using estimated marginal means (package ‘emmeans’) with Tukey correction for multiple comparisons [71]. To examine landscape effects within each infraclass (Marsupialia and Placentalia), we conducted separate binomial GLMs per infraclass. To assess differences in prevalence between protected and unprotected areas, we used a Chi-square test. We also tested for a correlation between trypanosome prevalence and species diversity in unprotected versus protected study sites using Spearman correlation using the cor.test() function from the R base package ‘stats’ [72]. Finally, we used a generalized linear mixed model (GLMM) with the glmer() function from the ‘lme4’ package to analyse the probability of trypanosome infection in each small mammal community, as well as specifically in *Proechimys semispinosus*, with infection status coded as a binomial response variable (1 for positive, 0 for negative) and capture site as a random effect, using a binomial distribution with logit link [73]. The explanatory variables included marsupial density, density of *Proechimys semispinosus*, and Shannon species diversity. For the *Proechimys semispinosus* subset, we incorporated the mean genetic diversity of the host species per capture site. All models underwent a multicollinearity assessment using the check_collinearity() function in the ‘performance’ package [74]. In every case, the predictor variables displayed minimal correlation, with VIF factors below 3. *P*-values were reported for all GLMMs in the results section, with a significance level set at alpha = 0.05.

## 3 Results

### 3.1 Trypanosome screening and variation in susceptibility across species

**Fig. 2:**
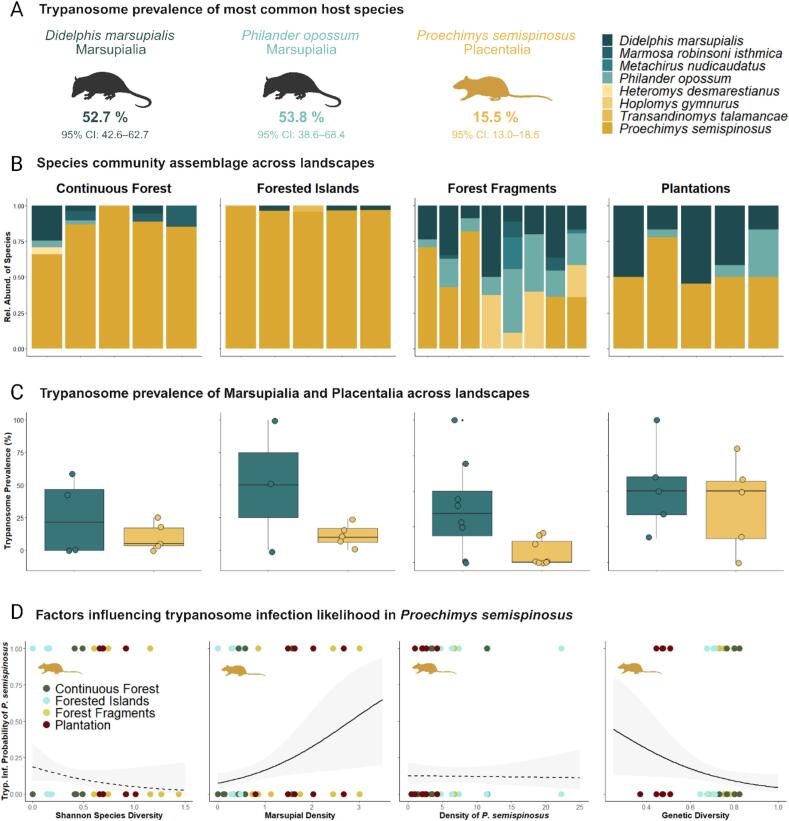
Community composition and determinants of *Trypanosoma cruzi* infection likelihood across hosts and landscape types. Trypanosome prevalence of the most common host species in the study area in central Panama (A). The relative composition of the species community per capture site (B) and trypanosome prevalence of marsupial and placental mammals (C) across the landscape types. (D) shows the trypanosome infection probability of *Proechimys semispinosus* in relation to Shannon species diversity, marsupial density and host genetic diversity, based on generalized linear mixed effect model predictions. Solid and dashed lines represent significant and non-significant fitted model curves, respectively, and lightly shaded area the respective 95 % confidence interval. Icons were created with BioRender.com.

In the Panama Canal area, a total of 830 small mammals were live-trapped at 23 different study sites spread across four landscapes with varying degrees of human disturbance and fragmentation (Fig. 1A and B), and were tested for *Trypanosoma cruzi* infections. The 830 small mammals were categorized into the two infraclasses, Marsupialia and Placentalia. Of these, 174 were found to be infected with trypanosomes. These infections were unevenly distributed among eight distinct species, highlighting significant variations in infection rates among different animal species (Fig. 2A). Among the generalist marsupials, *Didelphis marsupialis* (*n* = 48/91; 52.7 %, 95 % CI: 42.6–62.7) and *Philander opossum* (*n* = 21/39; 53.8 %, 95 % CI: 38.6–68.4) exhibited the highest prevalence of infection, while the generalist rodent species *Proechimys semispinosus* (*n* = 103/664; 15.5 %, 95 % CI: 13.0–18.5) showed a lower overall prevalence. Additionally, we detected one positive trypanosome case in the rare rodent species *Heteromys desmarestianus* (n = 1/2; 50.0 %, 95 % CI: 9.5–90.5). Conversely, for the other relatively rare marsupial and rodent species, including *Marmosa robinsoni isthmica* (*n* = 0/16), *Metachirus nudicaudatus* (n = 0/3), *Hoplomys gymnurus* (n = 0/14), and *Transandinomys talamancae* (n = 0/1), there was no evidence of *Trypanosoma cruzi* infection based on the low number of tested animals. These findings underscore the substantial variation in trypanosome prevalence across host species (*p < 0.001), indicating differing levels of susceptibility to infection. In particular, the marsupials *Didelphis marsupialis* and *Philander opossum* exhibited high infection prevalence, whereas the spiny rat *Proechimys semispinosus*, the most abundant rodent species, showed lower prevalence compared to both marsupial species (*p < 0.001 for both pairwise comparisons).

### 3.2 Variation in trypanosome infection rates across the landscapes

Our findings revealed that trypanosome infections became increasingly prevalent with higher levels of disturbance (Fig. 1C), indicating a significant effect of land-use on trypanosome prevalence (*p < 0.001). Specifically, areas characterized by continuous forest exhibited the lowest prevalence (19.5 %, 95 % CI: 14.8–25.3). Forested islands, which are fragmented but otherwise protected and undisturbed, showed a slightly higher prevalence (12.9 %, 95 % CI: 9.7–16.8; *p* = 0.141). Prevalence increased further in forest fragments (27.1 %, 95 % CI: 21.3–33.8; *p* = 0.271) and peaked in monoculture plantations (49.3 %, 95 % CI: 37.7–60.9). Among the studied landscapes, monoculture plantations, marked by the highest degree of fragmentation and human disturbance, harbored a significantly higher trypanosome prevalence than continuous forest sites (*p < 0.001), forested islands (*p < 0.001), and forest fragments (*p = 0.006). While the prevalence among Marsupialia did not vary substantially across landscapes (Fig. 2C; range: 39.4–57.1 %, 95 % CI: 24.7–68.6, *p* = 0.332), our analysis revealed pronounced differences within the Placentalia group, which was primarily composed of the abundant rodent species *Proechimys semispinosus*. The overall prevalence in this group was around 15 %, but this increased to approximately 50 % in plantation areas (Fig. 2C; 47.4 %, 95 % CI: 32.5–62.7, *p < 0.001). In plantations, trypanosome prevalence among placental mammals was higher than in continuous forests (*p < 0.001), forested islands (*p < 0.001), and forest fragments (*p < 0.001). Prevalence in continuous forests, forested islands, and forest fragments showed no notable differences (range: 11.9–16.0 %, 95 % CI: 8.3–21.9, *p* ≥ 0.552).

### 3.3 Determinants of infection likelihood in *Proechimys seminspinosus*: influence of anthropogenic disturbances on species community assemblage and genetic diversity

The analysis of host community composition across various landscapes revealed a notable influence of anthropogenic impact on species assemblages (Fig. 2B). Specifically, anthropogenic disturbances led to a reshuffling of communities and clear variation in species composition (Fig. 2B, F_3,19_ = 2.285, R^2^ = 0.265, *p = 0.007). The mean density of marsupials varied across landscapes (F_3,19_ = 4.845, *p = 0.011), with forest fragments showing higher densities compared to forested islands (*p = 0.027). The mean density of spiny rats also differed across landscapes (F_3,19_ = 5.298, *p = 0.008), with forest fragments (*p = 0.008) and plantations (*p = 0.021) showing lower densities compared to forested islands. These shifts in community composition favored marsupial species such as *Didelphis marsupialis* and *Philander opossum*, which exhibited the highest infection rates, while the densities of the alternative rodent host *Proechimys semispinosus* declined.

Building on these findings, our analysis aimed on identifying the factors influencing the probability of trypanosome infection in the spiny rat *Proechimys semispinosus*. Specifically, we examined the roles of species diversity, the densities of marsupials and *Proechimys semispinosus*, and the genetic diversity of the rodent host itself (Fig. S1 and Schwensow et al., 2022 [59]). Marsupial density was positively associated with spiny rat infection probability (Fig. 2D and Table 1, *p = 0.014), whereas host genetic diversity showed a negative association with infection likelihood (Fig. 2D and Table 1, *p = 0.039). Species diversity (Fig. 2D and Table 1, *p* = 0.150) and the density of the rodent host itself (Fig. 2D and Table 1, *p* = 0.874) were not associated with infection probability.

**Table 1:** Effects of community and genetic factors on *Trypanosoma cruzi* infection probability in *Proechimys semispinosus*. Summary table of the results from a binomial generalized linear mixed model with logit link function, examining the effect of Shannon species diversity, host density (marsupials and rodents), and host genetic diversity on trypanosome infection risk of *Proechimys semispinosus*. Significant results are in bold.

	*Predictors*		*Odds Ratios*	*CI*	*p-Value*
Trypanosome infection likelihood in *P. semispinosus*	Trypanosome infection ∼ Shannon Species Diversity + Marsupial Density + Genetic Diversity of *P. semispinosus* + (1|Capture Site)				
	(Intercept)		2.15	0.15–30.42	0.573
	Shannon Species Diversity		0.23	0.03–1.71	0.150
	Marsupial Density		2.46	1.20–5.03	**0.014**
	*P. semispinosus* Density		0.99	0.93–1.06	0.874
	Genetic Diversity		0.02	0.00–0.83	**0.039**
	Random Effects				
	σ2	3.29			
	τ00 capture_site	0.43			
	ICC	0.12			
	N capture_site	20			
	Observations	663			
	Marginal R^2^ / Conditional R^2^	0.087 / 0.192			

### 3.4 Trypanosome prevalence and infection likelihood: The impact of conservation status

**Fig. 3:**
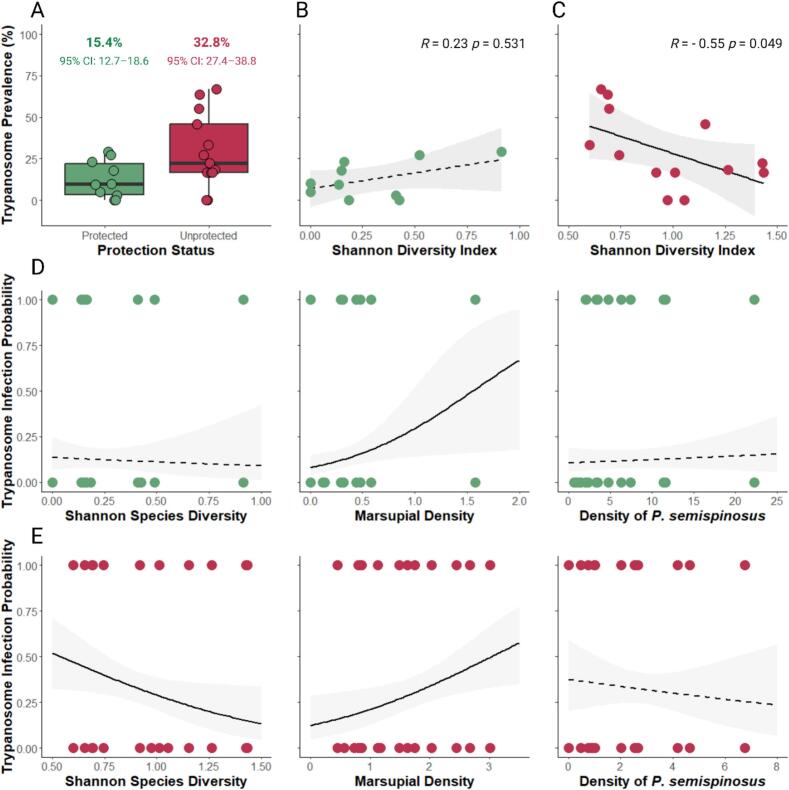
Trypanosome prevalence and infection likelihood in relation to community characteristics and protection status. Trypanosome prevalence in protected versus unprotected study locations (A). Each data point in the boxplots represents prevalence at an individual capture site, while the values above indicate overall prevalence per protection status, with 95 % confidence intervals. Spearman correlation between trypanosome prevalence and Shannon species diversity in protected sites (B) as well as unprotected sites (C). The likelihood of trypanosome infection in individuals within species communities at protected (D) and unprotected (E) sites in relation to Shannon species diversity, marsupial density and host genetic diversity. Solid and dashed lines represent significant and non-significant fitted model curves, respectively, and lightly shaded area the respective 95 % confidence interval.

Additionally, our investigation into the relationship between trypanosome prevalence and conservation status (protected versus unprotected study sites) revealed clear differences. Unprotected areas showed a higher prevalence of trypanosome infections than protected areas (Fig. 3A; protected: 15.4 %, 95 % CI: 12.7–18.6, unprotected: 32.8 %, 95 % CI 27.4–38.8; χ^2^ = 31.53, df = 1, *p < 0.001). While the trypanosome prevalence was not correlated with Shannon species diversity in protected habitats (Fig. 3B; *r* = 0.23, *p* = 0.531), it showed a moderate negative correlation with Shannon species diversity (Fig. 3C; *r* = −0.55, *p = 0.049) in unprotected landscapes. Lastly, we investigated how community characteristics and species diversity influenced individual infection likelihood in relation to habitat conservation status. The models considered Shannon diversity, the density of marsupials, and the density of *Proechimys semispinosus*. In protected habitats, marsupial density (*p = 0.022) was positively associated with infection risk, while Shannon species diversity (*p* = 0.721), and the density of *Proechimys semispinosus* (*p* = 0.575) had no effect (Fig. 3D and Table 2). Conversely, in unprotected habitats, marsupial density (*p = 0.039) again showed a positive association, and higher Shannon species diversity (*p = 0.014) was linked to a lower infection risk (Fig. 3E and Table 2). The density of the less susceptible rodent host *Proechimys semispinosus* (*p* = 0.542) was not associated with infection risk.

**Table 2:** Influence of ecological factors on *Trypanosoma cruzi* infection likelihood in protected vs. unprotected sites. Summary of binomial generalized linear mixed effect models with logit link, analyzing the effects of Shannon species diversity and host densities on trypanosome infection likelihood in protected (continuous forest and forested islands) versus unprotected (forest fragments and monoculture plantations embedded in an agricultural matrix) sites. Significant results are in bold.

	*Predictors*		*Odds Ratios*	*CI*	*p-Value*
Protected sites	Trypanosome infection ∼ Shannon Species Diversity + Marsupial Density + Density of *P. semispinosus* + (1|Capture Site)				
	(Intercept)		0.08	0.03–0.20	**<0.001**
	Shannon Species Diversity		0.63	0.05–7.81	0.721
	Marsupial Density		4.79	1.25–18.37	**0.022**
	*P. semispinosus* Density		1.02	0.96–1.08	0.575
	Random effects				
	σ2	3.29			
	τ00 capture_site	0.20			
	ICC	0.06			
	N capture_site	10			
	Observations	570			
	Marginal R^2^ / Conditional R^2^	0.066 / 0.119			
Unprotected sites	Trypanosome infection ∼ Shannon Species Diversity + Marsupial Density + Density of *P. semispinosus* + (1|Capture Site)				
	(Intercept)		1.13	0.11–11.93	0.918
	Shannon Species Diversity		0.14	0.02–0.91	**0.039**
	Marsupial Density		1.91	1.14–3.20	**0.014**
	*P. semispinosus* Density		0.92	0.70–1.21	0.542
	Random effects				
	σ2	3.29			
	τ00 capture_site	0.16			
	ICC	0.05			
	N capture_site	13			
	Observations	259			
	Marginal R^2^ / Conditional R^2^	0.125 / 0.166			

## 4 Discussion

Human-induced alterations to the environment, such as modifications in land-use, stand out as the primary catalyst for the worldwide decline in biodiversity, carrying severe repercussions for wildlife health [19,75]. Our research in the Panama Canal region reveals a complex narrative about the intricate interplay between human activity, biodiversity changes—including shifts in species composition and genetic diversity—and their impact on disease dynamics. We document a significant increase in trypanosome prevalence associated with higher levels of anthropogenic disturbance. This rise is attributable to two main factors: a shift in mammalian community composition in disturbed landscapes favoring marsupial species, which exhibit the highest overall infection rates, and an increase in the prevalence of a less frequently infected rodent species, driven by the higher density of marsupial hosts and reduced genetic diversity among these animals. Overall, our results indicate that changes in species communities toward higher densities of generalist host species in human-altered landscapes may facilitate the spread of zoonotic pathogens.

The observed increase in trypanosome infection prevalence, from pristine forests to monoculture plantations, underscores the profound impact of habitat alteration on disease dynamics, with various landscape changes collectively contributing to this phenomenon. Essentially, landscape modifications, such as variations in the size of habitats, alterations in land cover, and shifts in resource availability, can lead to changes in the population densities of hosts and vectors, consequently influencing the risk of disease transmission [76]. The sharp rise in infection rates within monoculture plantations compared to natural forests underscores the impact of habitat simplification on disease transmission. Our study has identified key biotic changes driving altered trypanosome infection dynamics, with increased marsupial species density along the anthropogenic gradient indicating that these species benefit from landscape changes and demonstrate resilience to human disturbances. Species adaptability to environmental changes varies [77], with those thriving in altered environments adapting through phenotypic plasticity and genetic adjustments, influencing natural selection. Additionally, the absence of specialized predators in fragmented landscapes allows generalist species to proliferate [54]. Simultaneously, the two most abundant marsupial species, *Didelphis marsupialis* and *Philander opossum*, previously recognized as natural reservoirs for *Trypanosoma cruzi* [48,78,79], exhibited the highest infection rates among all species captured in our study. This observation is consistent with the frequently observed yet not entirely comprehended phenomenon that species displaying greater resilience to human disturbance tend to be high-quality hosts [28]. In principle, the differences in infection rates among marsupial and placental mammals, particularly between generalist species like *Didelphis marsupialis* and *Philander opossum* compared to *Proechimys semispinosus*, may not only reflect ecological factors but also diagnostic limitations: chronic-phase infections in placental mammals often result in low parasitemia, making them harder to detect through blood PCR [80,81]. In such cases, parasite confirmation may require tissue samples, particularly from cardiac tissue [82]. Nonetheless, the proliferation of marsupial species in these areas facilitates greater transmission opportunities and elevates the overall disease risk within these ecosystems, including for less susceptible host species, as seen in the increased prevalence of the rodent species *Proechimys semispinosus*. This pattern points to how environmental modifications can disrupt natural regulatory mechanisms that typically control pathogen spread. The positive correlation between marsupial density and trypanosome infection probability suggests that higher host densities, as a consequence of disturbed habitats, can facilitate more frequent contact between hosts and vectors. Although our study did not assess vector populations directly or include human infection data, the elevated prevalence in wildlife hosts in disturbed habitats aligns with findings from vector-focused studies. For instance, Moo-Millan et al. (2023) reported increased abundance and infection rates of *Triatoma dimidiata* in domestic habitats compared to sylvatic ones in rural Mexico [83]. This suggests that anthropogenic disturbance may simultaneously affect host and vector dynamics, amplifying transmission risks. The increased presence of susceptible hosts in human-impacted areas likely heightens exposure for livestock, pets, and humans, as domestic, peridomestic, and synanthropic animals such as rodents can bridge sylvatic and domestic cycles [47]. These dynamics highlight the importance of integrated disease surveillance across wildlife, vectors, and people, in line with the One Health framework. While our focus on small mammals has yielded key insights into host community roles in *Trypanosoma cruzi* transmission, this represents only part of the ecological complexity in these landscapes. Acknowledging that non-mammalian hosts and vector populations also play critical roles in transmission cycles [52,[84], [85], [86]], future research should adopt a broader ecological scope to fully capture the dynamics of *Trypanosoma cruzi* spread in disturbed environments.

The observed shift in species assemblage toward the dominance of competent hosts over non-competent species following human disturbance is a recurring pattern [23,25,87]. The consequences of these changes for disease dynamics depend heavily on the ecological traits and interactions within the host community [21,23,25]. Furthermore, in cases where less diverse assemblages coincide with higher abundance of competent hosts and fewer non-competent hosts, it can be challenging to disentangle the effects of changes in host abundance from intrinsic properties of biodiversity [21]. In a prior study, we found that habitat disturbance and fragmentation do not inherently lead to a decrease in species diversity [59]. Instead, when there is a significant amount of available habitat, as is the case with fragments or plantations surrounded by agricultural land, fragmentation is anticipated to increase species diversity, especially among highly mobile generalist species [59,88,89]. However, it is important to recognize that these disturbed and fragmented habitats likely experience a more extensive reshuffling of their ecological communities. Intriguingly, our study found that species diversity did not exert a significant influence on infection likelihood in protected habitats, while it does reduce infection risk in unprotected and disturbed habitats. This suggests that natural processes and barriers likely remain functional in protected habitats, contributing to ecological resilience and disease regulation [35]. Additionally, even in areas where land-use has changed and forests have been deforested, restoration and species conservation programs can play a crucial role in minimizing infection risks. This further underscores the importance of such initiatives in maintaining ecological balance and preventing disease spread.

Another important aspect of our findings lies in the observed decrease in host genetic diversity upon anthropogenic disturbance, which influences the likelihood of infection. This observation introduces a vital further dimension to the diversity-disease relationship. Specifically, the decline in genetic diversity even in a generalist species in more disturbed habitats implies a higher susceptibility risk to infection, shedding light on the hidden layer of biodiversity – genetic biodiversity – within host communities. Thereby, our study underscores the importance of considering genetic diversity within host populations as a critical factor in disease ecology. The emphasis on genetic diversity may hold a key to unraveling the complexities of the biodiversity-disease relationship [90]. In this context, our study highlights that the impact of anthropogenic disturbance on biodiversity at multiple levels can have cascading effects on disease dynamics.

## 5 Conclusion

Our study offers a nuanced understanding of the complex relationships among anthropogenic landscape changes, shifts in species assemblages and intraspecific genetic diversity, and infection risk. Our findings underscore the necessity of adopting integrated landscape management and conservation strategies that support both environmental sustainability and public health. In accordance with the One Health concept, we emphasize biodiversity at all levels, encompassing genetic diversity as a pivotal element in preserving ecosystem resilience and regulating disease dynamics. Recognizing and addressing the multifaceted impacts of habitat disturbance on pathogen transmission will be essential for safeguarding ecosystem integrity and protecting human and animal health. This integrated perspective is increasingly vital in an era where preserving the complexity of biodiversity is fundamental to global health security.

## CRediT authorship contribution statement

**Magdalena Meyer:** Writing – review & editing, Writing – original draft, Visualization, Methodology, Formal analysis, Data curation, Conceptualization. **Georg Eibner:** Methodology, Investigation, Formal analysis, Conceptualization. **Alexander Christoph Heni:** Methodology, Investigation, Formal analysis, Conceptualization. **Kerstin Wilhelm:** Methodology, Formal analysis. **Simone Sommer:** Writing – review & editing, Writing – original draft, Supervision, Project administration, Funding acquisition, Conceptualization.

## Funding sources

This research was funded by the German Science Foundation (DFG) and is part of the DFG Priority Program SPP 1596/2 Ecology and Species Barriers in Emerging Infectious Diseases (SO 428/9-1, 9-2 and DR 772/8-1).

Data and code availability.

The entire data and R code generated in this study have been deposited on GitHub (https://github.com/MagdalenaMeyer/Changes-in-Biodiversity-Drive-Trypanosoma-Infections-of-Wildlife-in-Panama).

## Declaration of competing interest

The authors declare no competing interests.

## Data Availability

The entire data and R code generated in this study have been deposited on GitHub (https://github.com/MagdalenaMeyer/Changes-in-Biodiversity-Drive-Trypanosoma-Infections-of-Wildlife-in-Panama).

## Appendix A Supplementary data

Supplementary material Image 1
