# Supplementary material for: Changes in biodiversity drive trypanosome infections of wildlife in Panama
Source: One Health. 2025 Jun 18;21:101113. doi: 10.1016/j.onehlt.2025.101113 (PMC12226096; doi:10.1016/j.onehlt.2025.101113)
Supplement: Supplementary file 1 — Supplementary material [file mmc1.docx]

Supplementary Materials for

**Changes in Biodiversity Drive Trypanosome Infections of Wildlife in Panama**

Magdalena Meyer* *et al.*

*Corresponding author. Email: magdalena.meyer@uni-ulm.de and simone.sommer@uni-ulm.de

This file includes:

Fig. S1


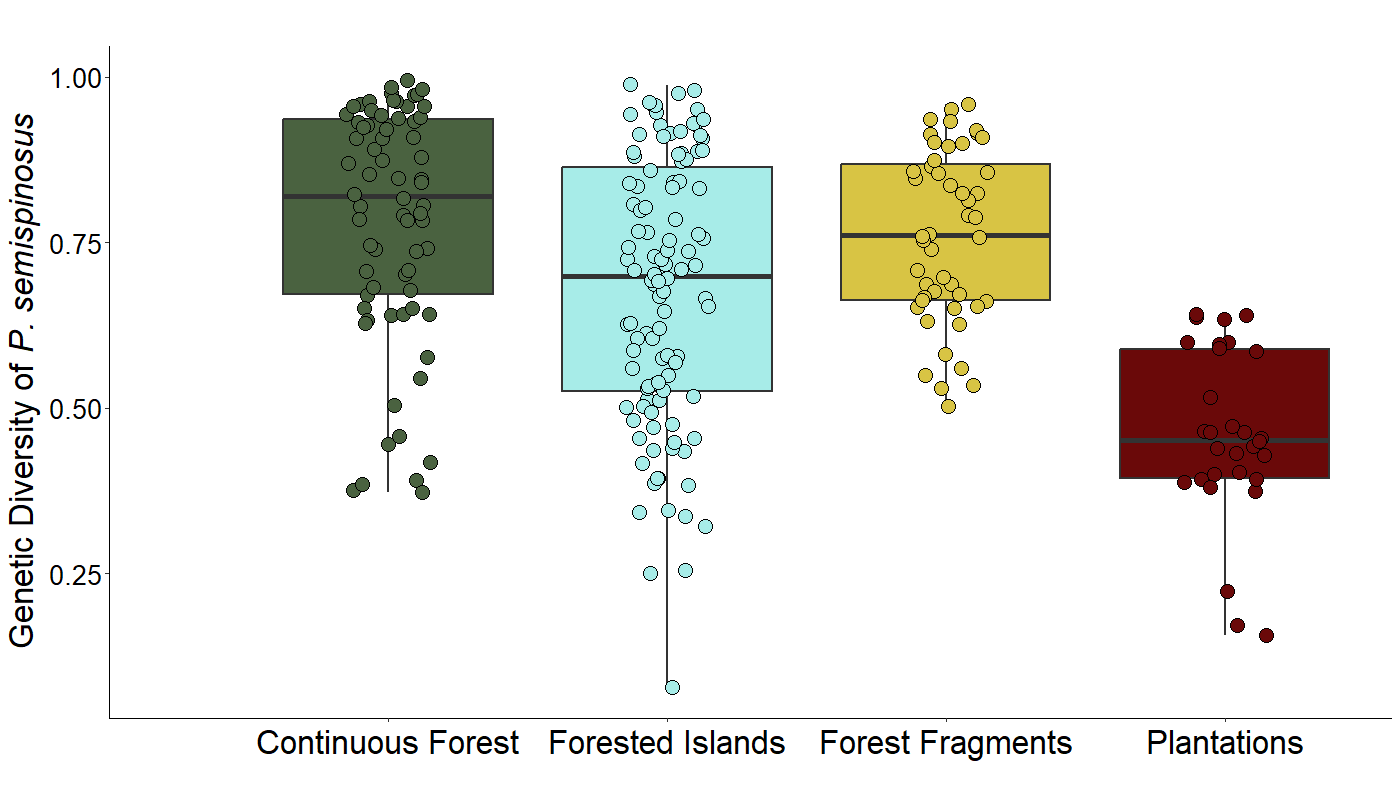


Fig. S1.

**Genetic Diversity of *Proechimys semispinosus* Across the Landscapes in Panama.**

Genetic diversity of *Proechimys semispinosus* across four landscape types: Continuous Forest (n = 108), Forested Islands (n = 70), Forest Fragments (n = 54), and Plantations (n = 30), with an overall sample size of 262 individuals. Each boxplot represents the distribution of genetic diversity within each habitat type, with higher values indicating greater genomic diversity. Points show individual measurements, highlighting variability across habitats with differing degrees of land-use and anthropogenic disturbance.
